# Multiple Stress Fractures in a Young Cancer Patient on Long-Term Zoledronic Acid: A Case Report and Review of Literature

**DOI:** 10.7759/cureus.69999

**Published:** 2024-09-23

**Authors:** Jiayi Weng, Ernest Beng Kee Kwek

**Affiliations:** 1 Department of Orthopaedic Surgery, Woodlands Health, Singapore, SGP

**Keywords:** atypical femoral fracture, bisphosphonate, fifth metatarsal fracture, metastatic cancer, stress fracture, subtrochanteric fracture, zoledronic acid

## Abstract

Bisphosphonates are commonly used in the treatment of osteoporosis and metastatic cancer patients with bone complications. Stress fractures are a well-known complication of long-term bisphosphonate treatment. Cancer patients receive much higher cumulative doses of bisphosphonates than osteoporotic patients and are subject to a higher risk of bisphosphonate-associated stress fractures. While there is an increasing number of reports of bisphosphonate-associated atypical femoral fractures (AFFs) and non-femoral stress fractures in osteoporotic patients, reports of such fractures in cancer patients are much rarer, especially non-femoral stress fractures. We present the first case report of an atypical subtrochanteric femur fracture following a sequential bilateral Jones fracture in a young non-osteoporotic patient with metastatic breast cancer after 12 years of zoledronic therapy. She sustained a left Jones fracture after seven years of zoledronic acid therapy and a right Jones fracture after 11 years of zoledronic acid therapy. She continued receiving regular zoledronic acid after these stress fractures and sustained a right subtrochanteric fracture after 12 years of zoledronic acid therapy. Both Jones fractures were treated conservatively, while the right subtrochanteric fracture was surgically fixed. Zoledronic acid was stopped after she sustained the AFF. This study highlights the need to look out for stress fractures beyond the commonly reported AFFs and atypical ulnar fractures when administering zoledronic acid to cancer patients.

## Introduction

Bisphosphonates are widely used in the treatment of osteoporosis to reduce the risk of vertebral and hip fractures. It is well known to be associated with complications such as atypical femoral fractures (AFFs) and non-femoral stress fractures [1-4]. Intravenous bisphosphonates, including zoledronic acid, are also the standard of care for cancer patients with bone complications, such as hypercalcemia of malignancy or osteolytic lesions. These patients receive much higher cumulative doses of bisphosphonates than patients with osteoporosis and are inevitably also subjected to the risk of bisphosphonate-associated stress fractures.

While bisphosphonate-associated stress fractures in osteoporosis patients are widely reported, there are only a few reports of such fractures in metastatic cancer patients with bone complications [5-8]. Herein, we report a patient with a right subtrochanteric fracture and metatarsal fractures while receiving zoledronic acid therapy for metastatic breast cancer.

## Case presentation

A 47-year-old woman was admitted after she tripped over an umbrella while getting out of the car and fell on her right hip. This led to acute right hip pain and inability to weight bear. She had been diagnosed with metastatic breast cancer 13 years ago and underwent lumpectomy and axillary clearance, followed by adjuvant chemotherapy and radiotherapy. She was on zoledronic acid for 12 years from 2012 to the time of admission. The dosage of zoledronic acid is 4 mg every 2 months from 2012 to January 2023 and 4 mg every 3 months from January 2023 onwards. She was also prescribed adjuvant hormonal therapy.

**Figure 1:**
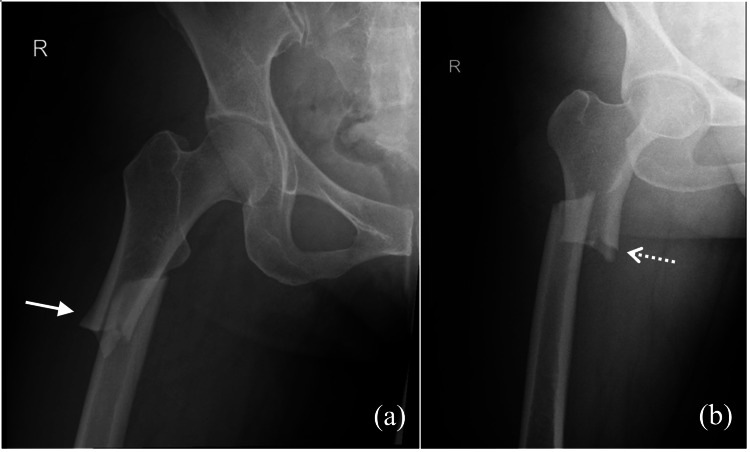
Radiographs of the right hip showing the transverse subtrochanteric fracture with lateral cortical thickening (solid arrow) and medial cortical spike (dotted arrow). (a) AP view and (b) lateral view

**Figure 2:**
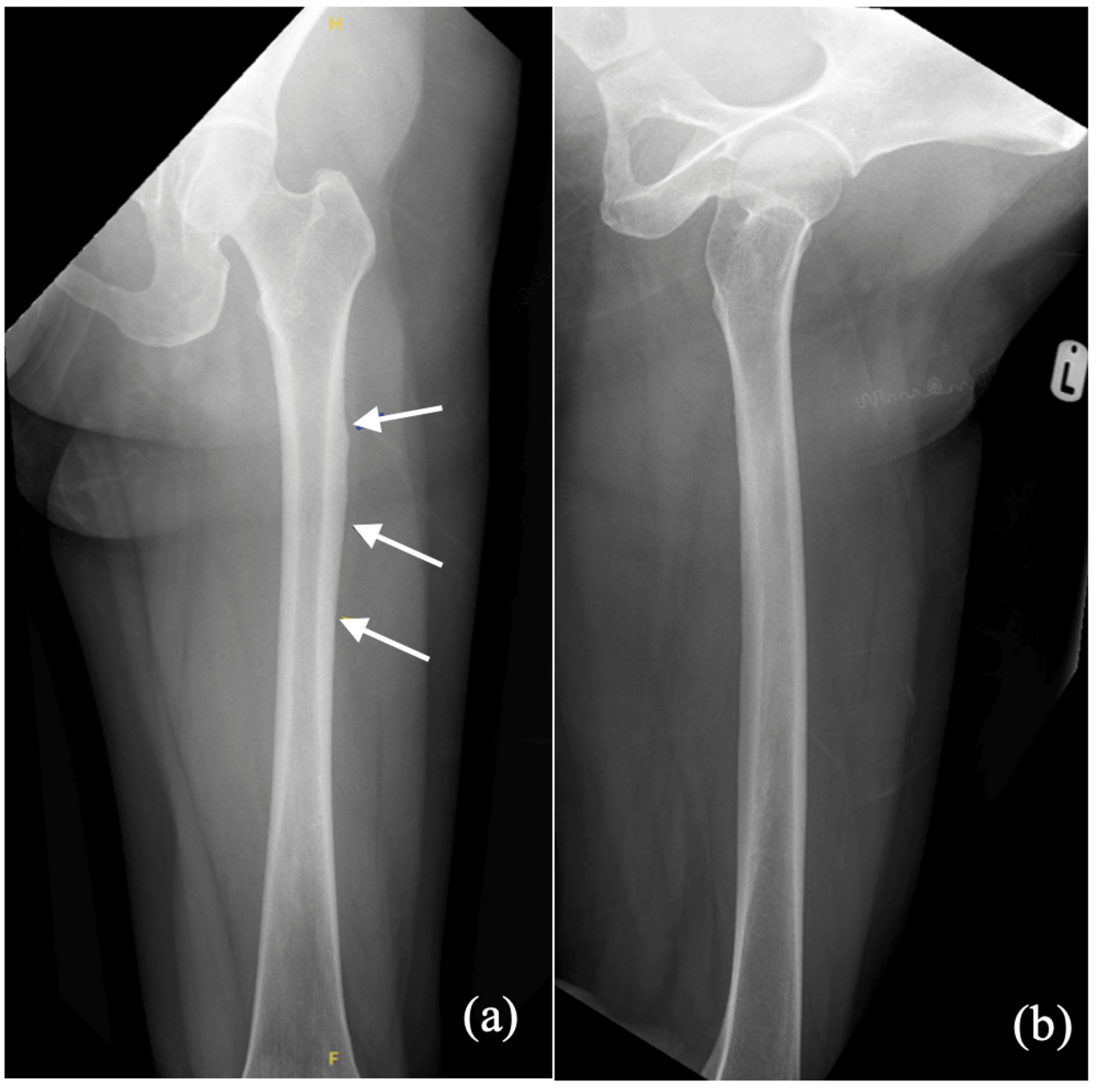
Radiographs of the left femur showing multiple periosteal reactions along the shaft (solid arrows). (a) AP view and (b) lateral view

Radiographs after admission showed a right subtrochanteric fracture (Figure 1) that demonstrated the classical findings associated with AFFs, as described by the American Society of Bone and Mineral Research (ASBMR) - lateral cortex thickening, transverse fracture, and medial cortex spike [9]. The contralateral femur showed multiple periosteal reactions along the shaft (Figure 2). Zoledronic acid was stopped in view of findings of lateral cortex thickening on the contralateral femur.

**Figure 3:**
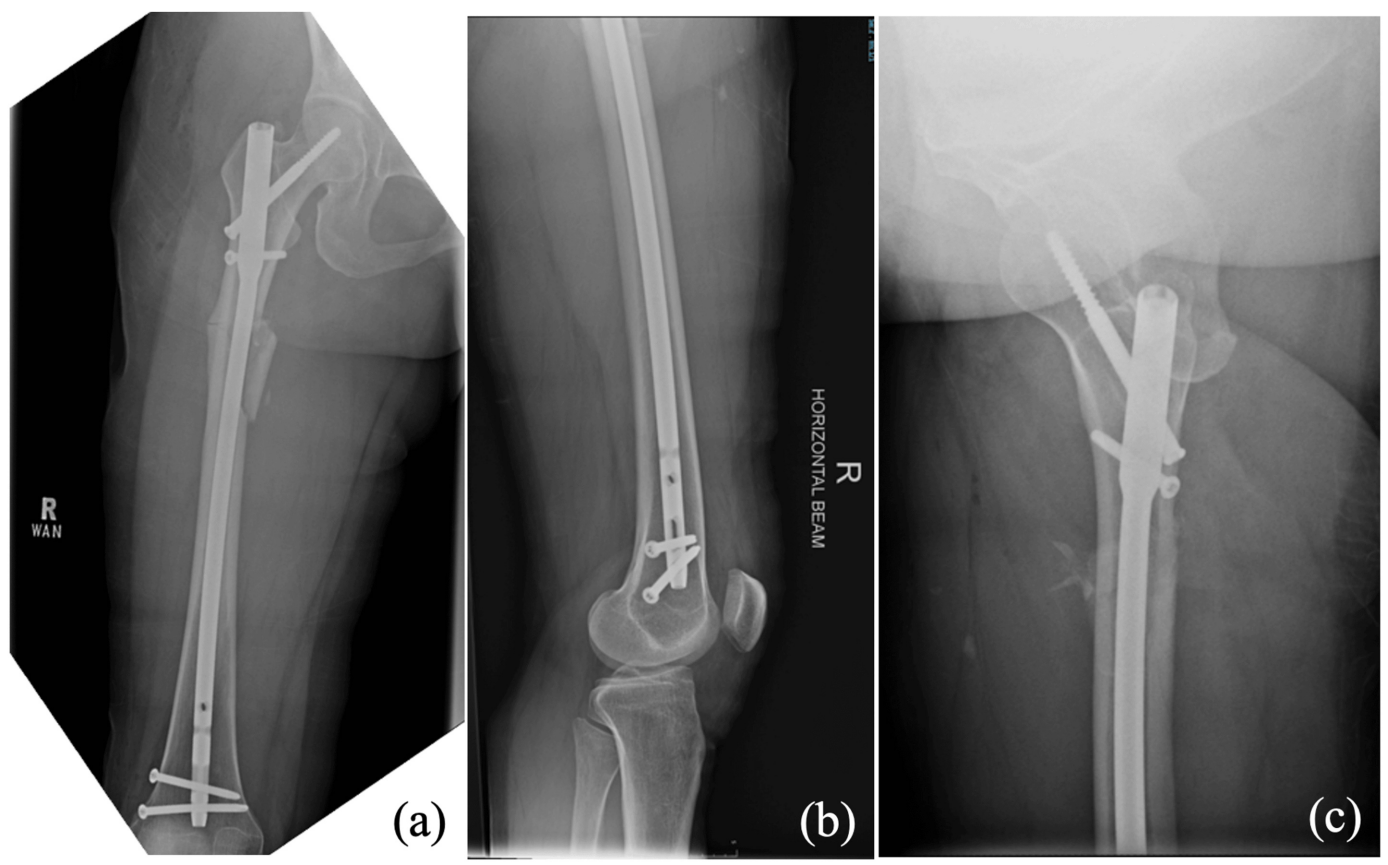
Radiographs of right femur postsurgery. (a) AP view, (b) distal lateral view, and (c) proximal lateral view

The patient underwent surgical fixation of the right hip with a femoral recon nail (DePuy Synthes, Raynham, MA) (Figure 3). Postoperatively, she was allowed full weight bearing to achieve earlier mobilization and started ambulating with a walking frame on postoperative day one.

**Figure 4:**
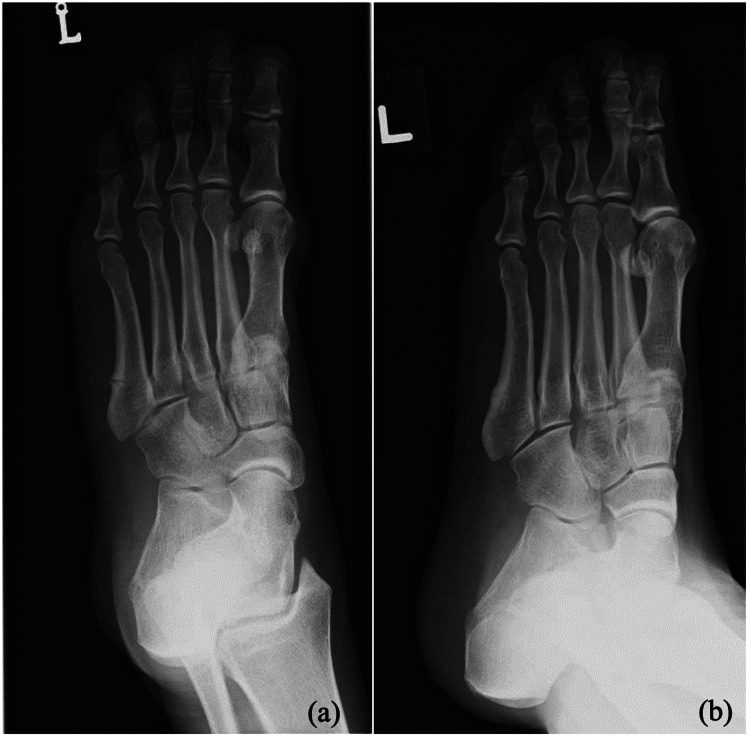
Radiographs of the left foot showing the Jones fracture in February 2019. (a) At initial presentation and (b) after six months

**Figure 5:**
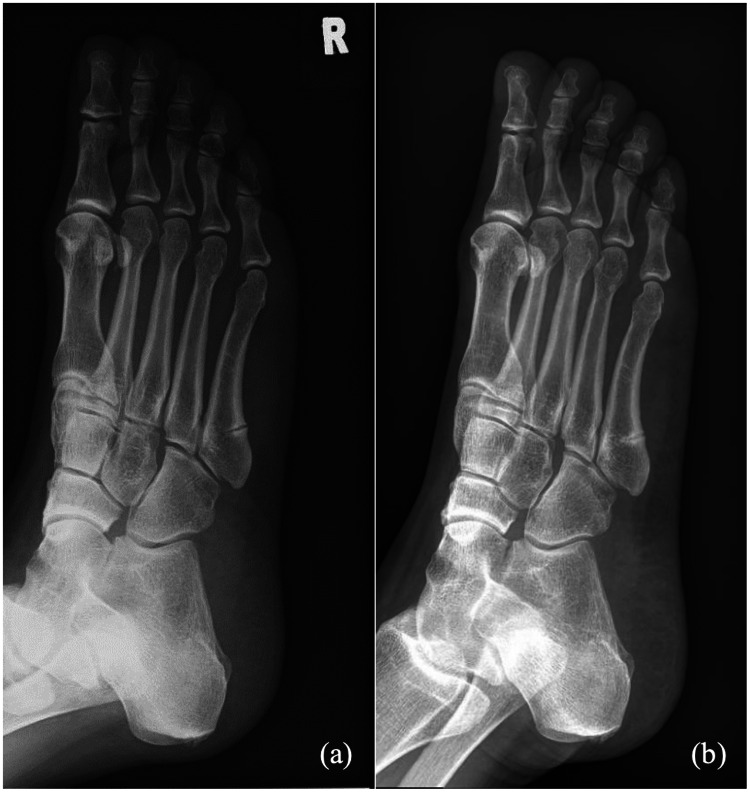
Radiographs of the right foot showing the Jones fracture in May 2023. (a) At initial presentation and (b) after six months

It was noted, during this admission, that the patient had also sustained bilateral sequential Jones fractures previously. Both were diagnosed as stress fractures at the initial presentation. She sustained a left Jones fracture (Figure 4) in February 2019, after being on zoledronic acid for seven years. The history of bisphosphonate use was overlooked by the care team at that time, and zoledronic acid was not stopped. She then sustained a right Jones fracture (Figure 5) in May 2023, after 11 years of zoledronic acid. Both were treated conservatively but had delayed union at six months postinjury. She also underwent a yearly bone mineral density scan while on zoledronic acid therapy, which showed normal range T scores.

## Discussion

Bisphosphonates are commonly used in the treatment of osteoporosis and have been shown to significantly reduce the rate of vertebral, hip, and other fractures among postmenopausal women with osteoporosis. Zoledronic acid is a third-generation bisphosphonate and has 10,000-fold to 100,000-fold greater antiresorption potency than the previous generations of agents [10]. The Health Outcomes and Reduced Incidence with Zoledronic Acid Once Yearly Pivotal Fracture Trial (HORIZON-PFT) showed treatment with zoledronic acid reduced the risk of morphometric vertebral fracture by 70% during a three-year period and reduced the risk of hip fracture by 41% [11].

In 2002, the US FOOD and Drug Administration approved an expanded indication for zoledronic acid that included its use in patients with metastatic breast cancer and myeloma. The American Society of Clinical Oncology recommends IV zoledronic acid (4 mg) every three to four weeks for patients with radiologic evidence of bone destruction [10]. Bisphosphonates reduce the risk of fracture, spinal cord compression, and hypercalcemia, thus improving overall survival outcomes in breast cancer patients with bone metastasis [12]. Studies also demonstrated that zoledronic acid can inhibit angiogenesis, invasion, and adhesion of tumor cells, thereby reducing bony metastases in cancer [13].

Despite the tremendous benefits of bisphosphonates, stress fractures related to severe suppression of bone turnover (SSBT) under bisphosphonate therapy are a well-known complication. Atypical femoral fractures (AFFs) are a type of stress fracture defined as a subtrochanteric transverse fracture with a characteristic appearance that occurs with low-energy or no trauma. The diagnosis criteria of AFFs are described by the American Society of Bone and Mineral Research (ASBMR): minimal/no trauma, transverse fracture line originating at the lateral cortex, medial spike, non/minimally comminuted, localized periosteal thickening of the lateral cortex at fracture site [9]. Kwek et al. described that more than half of patients with AFFs had bilateral findings of stress reactions or fractures [1]. Approximately 55-76% of the patients with bisphosphonate-related fractures had prodromal pain prior to fracture completion, whereas none of the patients with osteoporotic fractures in the absence of long-term alendronate had prodromal pain.

Cancer patients on bisphosphonates receive much higher cumulative doses than patients with osteoporosis, which puts them at higher risk of developing AFFs. Chang et al. reported four AFFs out of 39 patients with femur fractures and previous use of intravenous bisphosphonate for metastatic breast cancer [5]. Kim et al. reported a case of right subtrochanteric fracture in a metastatic breast cancer patient after four years of zoledronic acid therapy, with incomplete union of fracture four months postoperatively [6]. Kishimoto et al. also reported a case of right subtrochanteric fracture in a metastatic breast cancer patient after 3.6 years of zoledronic acid therapy; at five months postop, there was only slight callus formation at the medial femoral cortex [7]. Both of these case reports exhibited evidence of contralateral periosteal reactions, which was observed in our patient as well. Tomoaki et al. reported that three out of 18 AFFs in patients with bone metastasis on bone-modifying agents including zoledronic acid had failed to achieve bone union and required nonunion surgery; 11 AFFs had delayed union as they required much longer time to achieve union compared to ordinary AFFs [8]. Similar to osteoporotic patients, cancer patients on long-term bisphosphonate also have prodromal symptoms prior to fracture completion [8].

Besides AFFs, recent studies have reported other non-femur stress fractures associated with prolonged use of bisphosphonates in osteoporosis. Odvina et al. reported patients on bisphosphonate treatment for osteoporosis who sustained non-femur stress fractures including the humerus, tibia, lumbar vertebra, rib, sacrum, pubic rami, and metatarsal [3,14]. These atraumatic stress fractures developed after long-term alendronate treatment and exhibited evidence of delayed fracture healing. Other studies reported insufficiency fracture of the tibia [15], pelvis [16], and ulna [4,17-18] in osteoporotic patients on long-term bisphosphonate therapy.

Non-femur stress fractures associated with long-term bisphosphonate therapy are not exclusive to osteoporotic patients. In recent years, there have been a number of reports of atypical ulna fractures (AUFs) in cancer patients with bone metastasis on long-term bisphosphonate therapy [19,20], but a paucity of reports on other non-femur stress fractures. This study is the first case report of bilateral Jones fractures followed by an AFF in a young cancer patient without osteoporosis. Our patient had a left Jones fracture after seven years of zoledronic acid therapy and a right Jones fracture after 11 years of zoledronic acid therapy. She continued receiving regular zoledronic acid after these stress fractures and sustained the right subtrochanteric fracture after 12 years of zoledronic acid therapy. She did not have other risk factors for Jones fractures including intensive sports/work activities and pes cavus.

Management of bisphosphonate-associated stress fracture is especially complex in cancer patients. Discontinuing antiresorptives could potentially increase the risk of bone metastases or aggravate existing metastatic lesions. On the other hand, prolonged bisphosphonate treatment significantly increases the risk of stress fracture even in non-osteoporotic patients. While AFFs and AUFs are well reported, our study highlights the need to be vigilant for other stress fractures as well.

## Conclusions

We present a case of an atypical subtrochanteric femur fracture following a sequential bilateral Jones fracture in a young non-osteoporotic patient with metastatic breast cancer after 12 years of zoledronic therapy. This study highlights the need to screen for stress fractures when administering zoledronic acid to cancer patients without osteoporosis. This vigilance should be extended beyond AFFs and AUFs to include less common stress fractures. Judicious consideration should be taken to stop zoledronic acid after stress fractures have developed to reduce the risk of more debilitating fractures.
